# Depression and empathy in health professionals who work in the long-term care institutions for older adults

**DOI:** 10.1590/1980-57642021dn15-030011

**Published:** 2021

**Authors:** Marina Miranda Borges, Ana Julia de Lima Bomfim, Marcos Hortes Nisihara Chagas

**Affiliations:** 1 Research Group on Mental Health, Cognition and Aging, Universidade Federal de São Carlos – São Carlos, SP, Brazil.; 2 Department of Neuroscience and Behavior, Universidade de São Paulo – Ribeirão Preto, SP, Brazil.; 3 Instituto Bairral de Psquiatria – Itapira, SP, Brazil.

**Keywords:** depression, empathy, homes for the aged, aged, depressão, empatia, instituição de longa permanência para idosos, idosos

## Abstract

Empathy is an important factor to guarantee the quality of care provided in the long-term care institutions (LTCIs) for older adults, and depression is a factor that affects the health of the professional and, consequently, the care. Thus, it is important that studies are conducted on the relationship of these variables in this context.

**Objective:**

The aim of this study is to verify the relationship between empathy and depressive symptoms among health professionals working in the LTCIs.

**Methods:**

A cross-sectional study was carried out at LTCIs in the state of São Paulo, Brazil. The final sample was constituted by 101 health professionals (i.e., caregivers and nursing technicians) with direct participation in the care of institutionalized older adults. The instruments were used as follows: the Interpersonal Reactivity Index (IRI) to assess empathy and the Patient Health Questionnaire-9 (PHQ-9) for the diagnosis of depression. For the analyses, the patients were divided into groups with and without depression, according to the score of the PHQ-9.

**Results:**

The prevalence of depression among health professionals was 19.8%. Significant statistical differences were found between the groups for the total score of the IRI (p=0.029), for the emotional domain (p=0.023), and for the personal distress (p=0.009).

**Conclusions:**

The findings indicate that the presence of depression among health professionals at LTCIs is related to the higher levels of empathy, especially in the emotional domain. Thus, future studies that contribute to understanding how care must be provided with empathy, but without harming the health of the professional, should be carried out.

## RESUMO

A empatia é um importante fator na garantia da qualidade do cuidado prestado em Instituições de Longa Permanência para Idosos (ILPIs), e a depressão é um fator que prejudica a saúde do profissional e, consequentemente, esse cuidado. Dessa forma, é importante que se realizem estudos sobre a relação dessas variáveis nesse contexto.

**Objetivo:**

Verificar a relação entre empatia e sintomas depressivos em profissionais de saúde de ILPIs.

**Métodos:**

Estudo transversal, realizado em ILPIs no estado de São Paulo. A amostra final foi composta por 101 profissionais da saúde (cuidadores e técnicos de enfermagem), que participavam diretamente do cuidado de idosos institucionalizados. Os instrumentos utilizados foram: a Escala Multidimensional de Reatividade Interpessoal (EMRI) para avaliar empatia e o *Patient Health Questionnaire-9* (PHQ-9) para o diagnóstico de depressão. Para as análises, os participantes foram divididos em grupos com e sem depressão, de acordo com a pontuação do PHQ-9.

**Resultados:**

A prevalência de depressão entre os profissionais de saúde foi de 19,8%. Foram encontradas diferenças estatísticas significativas entre os grupos para a pontuação total do EMRI (p=0,029), para o domínio emocional (p=0,023) e angústia pessoal (p=0,009).

**Conclusões:**

Nossos achados apontam que a presença de depressão entre os profissionais de saúde de ILPIs está relacionada a maiores os níveis de empatia, principalmente no domínio emocional. Assim, pesquisas futuras que contribuam para entender como deve se dar o cuidado prestado com empatia, porém sem prejudicar a saúde do profissional, devem ser realizadas.

## INTRODUCTION

Currently, the long-term care institutions (LTCIs) for older adults have shown to be important as a supporting cornerstone for families that need help to provide care to older adults.^1^ Loss of functionality and cognitive impairment are frequent in part of the aged population and are the significant factors that can lead to their institutionalization, especially in the cases where the family cannot meet the demands derived from this process.^2^ ^,^ ^3^

In this context, the way in which care is provided to these older adults is the main point of discussion, and health professionals need to be technically and emotionally prepared for this role, with the development of empathy being essential in this process.^4^ By definition, empathy refers to the human ability to put oneself in someone else’s place and is divided into two main dimensions: (1) emotional/affective empathy, which is the appearance of feelings or emotions in response to someone else’s condition and (2) cognitive empathy, which refers to the ability to identify and understand the condition of another person.^4^

These health professionals, especially the nursing team, can be more susceptible to negative mental states, such as depression, anxiety, and stress, which can be related to some working conditions, such as workload and the need to work in shifts.^5^ In addition, the prevalence of dementia and depression in institutionalized older adults is high, i.e., a factor that causes increased dependence,^6^ which contributes to the fact that the professionals who provide care under these circumstances are at high risk of developing psychiatric disorders.^7^ The impairment of the mental health of these individuals can harm not only their own health but also the quality of the assistance provided.^5^

Faced with this situation of population aging and the need to provide quality care for older adults, this study aimed to identify the relationship between empathy and depressive symptoms among health professionals working in LTCIs.

## METHODS

### Setting and participants

This is a quantitative, cross-sectional, correlational, and observational study, with a convenience sample, carried out with health professionals working in LTCIs. The study sample consisted of the health professionals who directly participated in the care of institutionalized older adults. Nursing technicians and caregivers were included. According to the Brazilian legislation, nursing technicians are professionals with the qualification of high school and some technical training courses, and are linked to the Regional Nursing Council (Conselho Regional de Enfermagem – COREN).^8^ The caregivers of older adults need at least a completion of elementary school and a proven professional course or training. This last category is not linked to the COREN and corresponds to number 5162-10 of the Brazilian Code of Occupations (Código Brasileiro de Ocupações – CBO).^9^

It was decided not to include higher education professionals, considering that they do not spend much time with the older adults and that their weekly workload in the LTCIs is also lower.

In total, 34 LTCIs were invited to participate in this study, and only 20 accepted the invitation. Of the institutions participating in the study, 85% were private and 15% were philanthropic, and most of the resident older adults were partially dependent on the activities of daily life. Of these institutions, 23 professionals refused to participate. The final sample consisted of 101 health professionals, who were divided into groups with and without depression, according to the score of the Patient Health Questionnaire-9 (PHQ-9).

### Procedures

Initially, an invitation was made to the technical heads of the LTCIs. After acceptance, all caregivers and nursing technicians working in the institutions were invited to participate voluntarily. All the volunteers consented to their participation by signing the informed consent form, which was approved by the Research Ethics Committee at Universidade Federal de São Carlos (CAAE 81016717.0.0000.5504).

Subsequently, the professionals completed a sociodemographic questionnaire including information on age, gender, marital status, schooling, weekly workload, time in the profession, function, and if they work in more than one place. The data were collected during the professional’s shift, at the time chosen by the professional, and according to the day-to-day activities of each institution, so as not to disturb the work processes. The tests lasted around 30–45 min.

The following instruments were applied:

#### Patient Health Questionnaire-9

The PHQ-9 was used to assess the presence of depression.^10^ The test has nine items, and the answers can vary from 0 (i.e., “not at all”) to 3 (i.e., “nearly every day”) in each item, referring to the previous 2 weeks. The total score is based on the sum of the points assigned to each item, ranging from 0 to 27 points. The cutoff point considered for the presence of depression was a score greater than or equal to 10.^11^ ^,^ ^12^

#### Interpersonal Reactivity Index

The IRI, created by Mark Davis in 1983, was used to assess empathy.^13^ The scale assesses the affective/emotional dimension of empathy and the cognitive dimension, and it consists of three subscales, namely, Empathic Concern (EC), Personal Distress (PD), and Perspective-Taking (PT). The first two subscales, EC and PD, mentioned refer to the affective dimension of empathy, and the last one, PT, refers to the cognitive dimension. It consists of 21 items, and each subscale has 7 items. The general level of empathy is obtained by the sum of the scores of the three subscales, with the score for each item varying from 1 (i.e., “Does not describe me well”) to 5 (i.e., “Describes me very well”). In this scale, the higher the score, the greater the empathy. The scale used in this study was the version in Portuguese, validated by Koller, Camino, and Ribeiro.^14^

### Data analysis

The descriptive analysis was performed to characterize the sociodemographic profile of the groups. The Kolmogorov-Smirnov test was used to verify the normality of the data. The differences between groups were assessed by the Student’s *t*-test or by the Mann-Whitney U-test according to the sample distribution, and the χ^2^ test was used for the categorical variables. The Spearman’s correlation test was used to assess the correlation between the variables. The statistical analyses were performed using the *Statistical Package for the Social Sciences* (SPSS) statistical package, version 21.0. The significance level considered was p≤0.05.

## RESULTS

The prevalence of depression among the health professionals was 19.8%. Table 1 presents the sociodemographic data of the participants, stratified by the groups with and without depression. As expected, a significant statistical difference was found between the groups with and without depression in the PHQ-9 (p<0.001).

**Table 1.** Sociodemographic data of the participants, stratified by groups with and without depression.

	Group without depression (n=81)	Group with depression (n=20)	p-value
	Mean (SD)		
Age	36.54 (10.38)	37.45 (9.72)	0.724
Time in the profession (years)	5.53 (4.50)	5.41 (6.92)	0.344
Workload (h/week)	42.17 (2.91)	41.55 (4.40)	0.408
	**N (%)**		
Shift systems			
Fixed	20 (25)	2 (10)	0.154
Variable	61 (75)	18 (90)	
Gender			
Female	74 (91)	20 (100)	0.202
Male	7 (9)	0	
Marital status			
Single	35 (43)	11 (55)	0.669
Married	29 (36)	5 (25)	
Divorced	15 (19)	4 (20)	
Widow/widower	2 (2)	0	
Schooling			
0–9	14 (17)	4 (20)	
10–12	26 (32)	11 (55)	0.097
≥12	41 (51)	5 (25)	
Function			
Caregiver	39 (48)	14 (70)	0.065
Nursing technician	42 (52)	6 (30)	
Works in more than one place			
Yes	12 (15)	2 (2)	0.799
No	69 (85)	18 (18)	

SD: standard deviation; *statistically significant difference (p<0.05).

Table 2 shows the mean and standard deviation of the groups with and without depression in the IRI task and its respective domains. Significant statistical differences were found between the groups for the total score of the IRI task (p=0.029), for the emotional (p=0.023) and personal distress (p=0.009) domains.

**Table 2.** Mean and standard deviation of the groups with and without depression in the Interpersonal Reactivity Index task and its respective domains.

	Group without depression (n=81)	Group with depression (n=20)	Student’s *t*-test or *Z* score	p-value
	Mean (SD)			
IRI total	71.01 (±12.81)	79.42 (±12.91)	*t*_99_=-2.216	0.029*
Emotional domain	44.96 (±10.80)	51.78 (±9.72)	*t*_99_=-2.308	0.023*
Empathic concern	28.06 (±5.47)	29.42 (±4.40)	*Z*=-0.546	0.585
Personal distress	16.90 (±7.44)	22.36 (±8.35)	*Z*=-2.627	0.009*
Cognitive domain	26.04 (±4.99)	27.63 (±5.82)	*Z*=-1.380	0.168

SD: standard deviation; IRI: Interpersonal Reactivity Index; *statistically significant difference (p<0.05).

**Figure 1.**
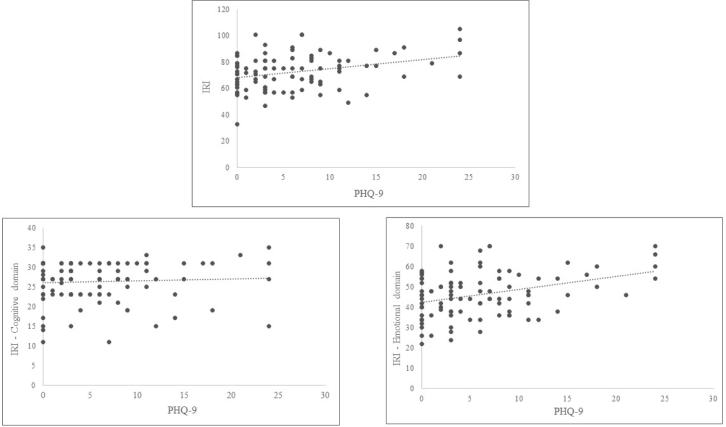
Scatter plot of correlation between PHQ-9 scores and the total IRI; between PHQ-9 and the emotional domain; and between the PHQ-9 scores and the cognitive domain.

IRI: Interpersonal Reactivity Index; PHQ-9: Patient Health Questionnaire-9.

In addition, weak positive correlations were found between the PHQ-9 scores and the total IRI scores (r=0.256; p=0.010), between the PHQ-9 and the emotional domain (r=0.293; p=0.002), and between the PHQ-9 and the personal distress domain (r=0.299; p=0.003). There was no correlation between the PHQ-9 scores and the cognitive domain (r=0.053; p=0.601) (Figure 1).

In relation to the presence of diseases, in the group without depression, 9% of the professionals reported systemic arterial hypertension; 2%, diabetes mellitus; 6%, cephalea; and 22%, low back pain. Among the professionals with depression, 5% reported arterial hypertension; 10%, cephalea; and 50%, low back pain.

## DISCUSSION

In this study, the prevalence of depression in caregivers and nursing technicians working in LTCIs was 19.8%, and the presence of depression was associated with a higher level of empathy, especially in the emotional domain. There were no differences between the groups in the other variables studied, including weekly workload and time working in the LTCI.

Despite the significant differences involved in care, recent studies show that the prevalence of depression in informal caregivers does not present significant differences when compared with the prevalence in formal caregivers, and the numbers found are close to what was identified in our study.^15^ ^-^ ^19^ In health professionals, the presence of depression can be a predictive factor for exhaustion and, consequently, can directly interfere in the quality of care provided to the patient.^20^

In relation to the care provided, according to Duarte et al.,^21^ although empathy is one of the main factors that lead to the quality of the care, if there is no balance, it can cause harms to the health of the professionals.^21^ In the study by Karanikola et al.,^22^ conducted with health professionals, a positive correlation was found between depressive symptoms and empathy, the results that corroborate with the findings of our study.^22^

The frequent use of the empathic capacity to deal with the patient’s suffering can cause a syndrome called “compassion fatigue,” still few studied in the Brazilian context.^21^ The exposure of the professional to stressful and traumatic events in relation to the patients can be a predictive factor for the onset of this syndrome, which leads to physical and emotional exhaustion in the professional, and which also presents rapid evolution.^23^ In the study conducted by Duarte et al.,^21^ it was found that the high levels of empathy in the emotional domain are a risk factor for compassion fatigue.^21^ In our study, we did not evaluate the presence of this syndrome, but it was found that the professionals who presented some indication of depression had higher levels of empathy in the emotional domain. In this way, we can infer that they could be more prone to the “compassion fatigue” syndrome.

Most of the studies involving actors who participate in the care of older adults are carried out with informal caregivers. In the study by Jütten et al.^24^ conducted with this category, a positive association was found between anxiety and affective empathy, and a negative association was found between depression and cognitive empathy.^24^ Although our sample has consisted of health professionals and not of informal caregivers, these results are similar to the findings of our study, as they show that affective empathy can be related to the appearance of negative emotions.

In the study carried out by Lee et al.,^25^ caregivers with higher levels of cognitive empathy assessed the task of caring as less stressful, were less depressed, and had greater satisfaction with life compared with caregivers with lower levels of cognitive empathy, while emotional empathy showed a negative correlation with life satisfaction.^25^ In this way, comparing these findings with the results of our study, we can conclude that cognitive empathy can be a protective factor for the mental health of the professional.

In the study carried out by Sampaio et al.^26^ with health professionals in the Brazilian context, positive associations were found between depression, anxiety, and one of the emotional components of empathy, namely, personal distress, also corroborating with the results of this study.^26^ Another finding of this study was that the use of cognitive aspects of empathy can prevent the onset of depression symptoms, reinforcing the possibility that empathy is associated with the mental health of the professionals who provide care.^26^

As a limitation of the study, it should be mentioned that only 20 LTCIs (58.8%) from the 34 that were invited participated in the survey. Although PHQ-9 is a widely used and validated instrument in different contexts, the use of structured or semi-structured clinical interviews is the gold standard for the diagnosis of depression; therefore, using the PHQ-9 to divide clinical groups with and without depression can be considered a limitation of this study.

In addition to that, considering that overload is a frequent condition within the care context and that it can result in the presence of the burnout syndrome, not evaluating this syndrome can be considered as a limitation since it shares similar characteristics of the mood disorders.

As a strength of this study, it can be highlighted that there are few studies addressing this theme in the Brazilian context. In addition, a large part of the national and international studies related to the care of older adults have informal caregivers as samples, not health professionals, i.e., individuals who have played an important role in the provision of care to older adults due to population aging and to the increase in the number of LTCIs. Thus, this study contributes to the research of empathy and depression in the institutional context, enabling the planning of actions aimed at the quality of the care provided, also paying attention to the health of the professional.

We observed a high prevalence of depression in health professionals of LTCIs. In addition, empathy, especially the affective domain, seems to be a factor to be considered in the evaluation of the mental health of caregivers and nursing technicians working in LTCIs.
